# Digitalization of prevention and treatment and the combination of western and Chinese medicine in management of acute heart failure

**DOI:** 10.3389/fcvm.2023.1146941

**Published:** 2023-05-25

**Authors:** Yingxin Wang, Herong Cui, Liwen Li, Yajing Cao, Hanyun Qu, Halisi Ailina, Zhili Dou, Chuwei Tang, Wanli Qin, Chenlu Wang, Xue Yang, Shixing Feng, Yixing Liu, Dongran Han

**Affiliations:** ^1^ School of Life Science, Beijing University of Chinese Medicine, Beijing, China; ^2^ School of Acupuncture and Moxibustion and Tuina, Beijing University of Chinese Medicine, Beijing, China; ^3^ Department of Biostatistics, School of Public Health, Peking University, Beijing, China; ^4^ Dongfang Hospital, Beijing University of Chinese Medicine, Beijing, China; ^5^ School of Management, Beijing University of Chinese Medicine, Beijing, China

**Keywords:** digital medicine, acute heart failure, combination of Chinese and western medicine, Chinese medicine, western medicine

## Abstract

Digitalization has emerged as a new trend in healthcare, with great potential and creating many unique opportunities, as well as many challenges. Cardiovascular disease is one of the major causes of disease-related morbidity and mortality worldwide, and the threat to life posed by acute heart failure is evident. In addition to traditional collegiate therapies, this article reviews the current status and subdisciplinary impact of digital healthcare at the level of combined Chinese and Western medical therapies. It also further discusses the prospects for the development of this approach, with the objective of developing an active role for digitalization in the combination of Western and Chinese medicine for the management of acute heart failure in order to support maintenance of cardiovascular health in the population.

## 1. Introduction

Digital medicine is a new method of medical treatment that involves the application of modern computing technology and information technology to medical treatment. China has gradually begun to apply computing technology in the context of hospitals since the 1960s, and the use of digital medicine has gradually evolved from a low level, involving the use of simple applications, to one of great breadth and depth. The use of digital tools upgrades the practice of medicine to a high-definition and more personalized level. A smartphone-centric approach enables each individual to generate real-world data and pay more attention to their health (1). With the development of AI technology, AI is transforming electrocardiograms (ECGs) into screening tools and predictors of cardiac and non-cardiac disease through the discovery of common subclinical patterns in very large data sets, often in asymptomatic individuals, without hard-coded rules (2). AI can effectively interpret the information in the ECG, and can thereby provide significant help in rapidly suggesting effective diet, exercise, and maintenance interventions, identifying potential disease hazards, and enhancing the daily protection of patients. On the other hand, the retrieval of past information is a new problem, since patients' activity trajectories are not constant, different regional databases are not connected, and data can be lost due to system updates. The implementation of a consistent system of digital records and medical history-taking will undoubtedly improve the diagnosis and treatment of diseases. At the same time, duplication of tests or delays in the diagnostic or treatment process can be avoided. Ultrasound, endoscopy, tomography, and pathology/histology already offer considerable digital potential, such as AI-supported diagnosis and treatment of visceral diseases. This helps with standardization, improvement of the detection of pathological findings, and acceleration of the diagnosis (3).

Acute heart failure (AHF) is becoming a global epidemic due the high morbidity, mortality, and cost of treatment associated with it (4). Hospital admission for acute heart failure is the most common cause of hospitalization in patients over 65 years of age. Now that population aging is a global mega-trend, acute heart failure is bound to become a global public health burden. AHF is associated with significant multi-organ dysfunction, and particularly with worsening renal function and ultimately failure, which leads to a long clinical course for AHF patients and prolonged hospitalization of patients; the management of the acute phase also poses a challenge to hospital systems. A great deal of effort is required to achieve good outcomes. Heart failure patients have nurses who closely monitor changes in their condition during hospitalization, and overall treatment compliance is high. However, after discharge, inadequate disease awareness, along with untimely treatment and index testing, predisposes patients to improper disease management, resulting in repeated admissions and a poor prognosis. Acute heart failure can be triggered again by factors external to the patient or by their own emotions or labor, so it is also necessary to maintain and observe all the patient's basic data indicators. Therefore, certain apps and sports watches used for health monitoring and medication reminders play a very prominent role in the lives of heart failure patients. They can detect and record the patient's heart rate, blood pressure, and sleep quality, which is beneficial for the patient's self-regulation and provides a more comprehensive data record for reference by their doctors. However, although smartphones and watches can measure blood pressure, the reliability of the data is not yet accepted by doctors for analysis of the patient's condition. One way to address these serious shortcomings is to embed multimodal sensors in household appliances to enable the collection of information on physical activity, sleep quality, and vital signs in daily life (5). Acute heart failure has an acute onset, and once a patient is encountered with sudden onset of the disease, the time available for effective resuscitation is a matter of seconds. Efficient and timely resuscitation depends on a sound and effective emergency medical system. The modern emergency medical system consists of pre-hospital emergency management, the hospital emergency department, and monitoring rooms. The application of a dedicated network in hospitals helps to quickly transfer the data collected by wireless monitoring equipment to the hospital. The patient's consultation records can be accessed through the database, which is helpful in enabling experts to judge the patient's condition and significantly reduces the time and cost of emergency medical treatment.

Currently, conventional Western medical treatment combined with Chinese medicine can effectively improve cardiac insufficiency, clinical symptoms, and quality of life in patients with acute heart failure. There is an urgent need to form clinical guidelines for the treatment of heart failure that can be followed and repeated, especially given the significant advantages of remote monitoring in the management of patients with acute heart failure. There is an urgent need to apply health programs and other digital medical technologies in order to adjust the existing Chinese and Western healthcare models and thus optimize clinical treatment protocols (6). Based on this view, this article summarizes the current status of the corresponding research and the main bottlenecks in integrating digital healthcare for acute heart failure with combined Chinese and Western treatments. We also draw out the prospects and potential of the use of digital prevention and treatment with combined Chinese and Western medicine in acute heart failure, in order to provide a basis for the proposal and development of new strategies and protocols for the clinical management of acute heart failure.

## 2. The current state of digital healthcare in acute heart failure

**Figure 1:**
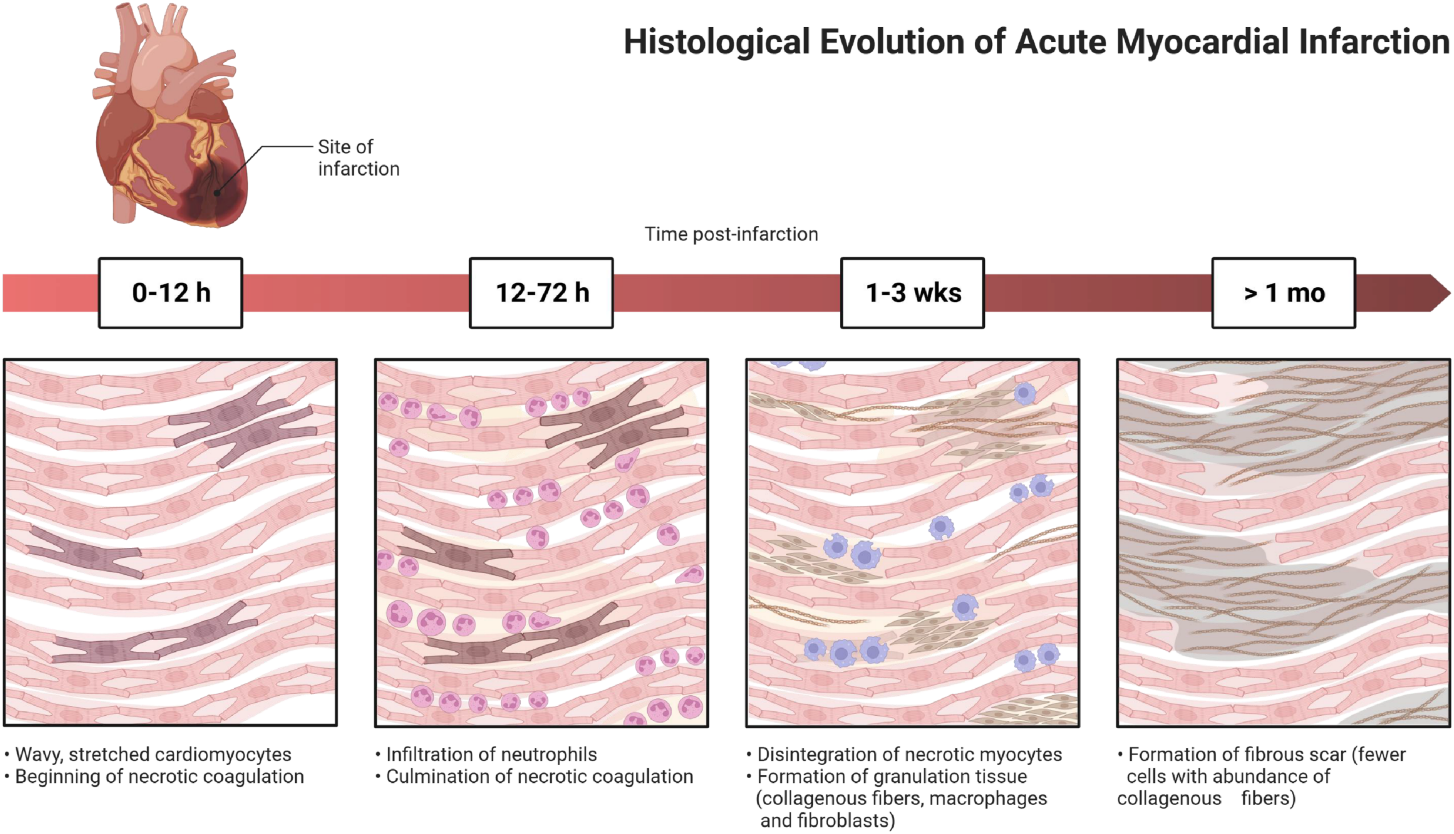
Demonstrates the importance and necessity of prevention and treatment by showing the progress of their disease.

Cardiovascular disease is the leading cause of morbidity and mortality worldwide, and up to half of heart failure patients die within 5 years of first diagnosis (7), putting enormous pressure on healthcare budgets and insurance premiums (8). A number of studies have shown that through the use of telecontrol, telemedicine, and wearable intelligent devices for heart failure patients, along with the use of various methods, such as cell phones, to detect the patient's clinical status and the stability of their condition, a 20% reduction in all-cause mortality can be achieved. Additionally, according to a meta-analysis, the positive effect on the condition produced by the use of telecontrol and telemedicine at home is likely to be dependent on timing, with the effect showing an enhanced trend from 0 to 6 months and a diminishing trend from 6 months to 12 months (Figure 1), as well as the relevance of factors such as weight, blood pressure, and other vital signs and psychological characteristics (9). Analyses can be conducted on the data collected in order to draw conclusions or to model diseases and patients; these methods can also provide support for further digitization, i.e., in the field of artificial intelligence.

With the availability of more data dimensions and a greater focus on modeling, machine learning and deep learning have also been gradually increasing in use in recent years for algorithms such as prediction and identification (10). As mentioned previously, most of the data sources for these models come from clinical cases, records, and data generated by telemedicine and wearable devices. The aim is primarily to detect and accurately identify patients who need interventions to reduce mortality (11). The algorithms used include neural networks, random forest algorithms, and logistic regression (12). These can be used to validate original models and to generate new models based on relevant studies (13), thereby having an enormous impact on population health. At the same time, various studies also recognize that this technology has great potential and high upsides.

## 3. Current status of combined Chinese and western medical therapies for acute heart failure

### 3.1 General situation

#### 3.1.1 Preparations and ready-to-use medicines

Heart failure is a disease characterized by weakness, palpitation, wheezing, and edema of the limbs. In clinical practice, the primary treatment method is to enhance Qi, nourish Yin, warm Yang, and promote water, and the prescriptions often used are Zhen Wu prescription, Si Wei Renshen prescription, Shengmai San, etc. The final medicine is often administered in the form of a Ganjiangfuzi prescription, Tianwangbuxin Dan, or Shexiangbaoxin pill.

Lu X et al. observed the effects of a Ganjiangfuzi prescription on cardiac function and plasma biochemical indices in a rat model of acute heart failure; they found that the administration of Ganjiangfuzi prescription at different concentrations caused a dose-dependent increase in + dP/dtmax and also an increase in -dP/dtmax, suggesting that Ganjiangfuzi prescription has the effect of maintaining and improving cardiac function in rats with acute heart failure (14). In terms of biochemical indexes, CK, LDH, BNP, and cTnT were reduced to a greater extent in the plasma of rats in the Ganjiangfuzi prescription group, and this group showed concentration-dependent effects in comparison with other groups, using a control group as reference. This finding suggests that the efficacy of Ganjiangfuzi prescription is more significant in acute heart failure compared with that of the Western drug digoxin and the application of Fuzi or Ganjiang alone.

Fu J et al. divided the rats used in the model of heart failure into three groups: two experimental groups were administered Qiliqiangxin capsules and Benadryl at appropriate concentrations, and the control group was administered equal amounts of saline by oral gavage (15). Efficacy observations indicated that both interventions significantly improved the biochemical indices of heart failure, among which, the effect of Qiliqiangxin capsules on AngPRL-4 was more substantial than that of Benazepril. In addition, the two experimental groups also exhibited effects on the potential metabolic markers of heart failure, with Qiliqiangxin capsules being more effective than Benazepril in regulating the metabolism of lipoxides and LPA.

#### 3.1.2 Injections

Chinese herbal injections are an extension and development of tonics and have been used for more than seventy years (16). Because of their rapid therapeutic effects, various types of injections are widely used in clinical practice today (17).

Renshen and Maidong are widely used in these injections. Modern pharmacological studies have shown that Renshen has the effect of inhibiting the activity of Na + -K + -ATPase, promoting Ca2 + inward flow, enhancing myocardial contraction, and improving cardiac function (18). The active ingredient in Maidong is Maidong polysaccharide, which protects cardiomyocytes, improves blood flow, and is able to increase free radicals and remove oxygen free radicals produced by myocardial ischemia, thereby dilating the coronary arteries and increasing blood flow there.

In Li M et al., patients with acute heart failure were allocated to two groups, with the treatment group being administered a Shenfu injection combined with levosimendan infusion and the control group being administered only levosimendan infusion (19). Efficacy was greater for the treatment group than for the control group, with the treatment group showing more significant improvement in blood pressure and hemodynamic parameters.

Wang SM et al. divided patients falling into cardiac functional classes II-IV into three groups: a group treated with Shenmai injection drip, a group treated with trimetazidine drip, and a group treated with conventional heart failure medication (control group) (20). Hu SY et al. used salt-sensitive rats to construct a hypertensive heart failure model, and divided such rats into three groups: a control group, a pirfenidone group, and a Shenmai injection group (21). The results showed that the left ventricular ejection fraction (LVEF) and left ventricular fractional shortening (LVFS) were more effectively increased among rats with heart failure in the Shenmai injection group than among those in the control group, and an improvement in myocardial morphology and a significant decrease in collagen volume fraction were observed in section staining (*P* < 0.05). Additionally, expression of type I collagen was reduced in the rats in the Shenmai injection group, suggesting that Shenmai injection inhibited myocardial fibrosis induced by hypertension.

Most existing studies have found positive effects in terms of the efficacy of TCM (traditional Chinese medicine) in the treatment of acute heart failure, with significant improvements to biochemical parameters and hemodynamics. It should be noted that the number of studies available in the literature for inclusion is small, so further research is needed on the topic of combined Chinese and Western medicine for the treatment of acute heart failure. We hope that more relevant clinical trials will emerge in the future.

### 3.2. Special implications in pediatrics and emergency care

#### 3.2.1. Current status of combined Chinese and western medicine in the treatment of pediatric acute heart failure

Acute heart failure is a common acute and critical illness in pediatrics. A number of recent experiments have involved systematic use of a combination of Chinese and Western medicine in order to observe the effects of this treatment and compare it to treatment with Western medicine alone; it has been found that treatment with a combination of Chinese and Western medicine has better efficacy in this disease.

In one study, a control group was administered routine treatment with cardiotonics, diuretics, vasodilators, and other Western drugs, while a treatment group was treated with Chinese medicine formulas according to the diagnosis and treatment, with the inclusion of different Chinese medicines according to various strands of evidence; the principle of medication was always the same. The finding was that the cure rate of the treatment group was significantly higher than that of the control group, and the onset of action days was also significantly shorter in the treatment group than in the control group. This indicates that a combination of Chinese and Western medicine for treatment of acute heart failure in infants and children is more effective and quicker than Western medicine alone (22).

Some experiments have shown that the addition of Shenmai or Shenfu injection to a foundation of conventional treatment with Western drugs (such as cardiac stimulants and diuretics) can reduce the dosage of cardiac glycosides, alleviate their toxic side effects, and shorten the amount of time needed to correct the issue in cases of pediatric acute heart failure, achieving better efficacy (23). In cases of pediatric pneumonia combined with heart failure, combined treatment with Chinese and Western medicine is more effective than Western medicine alone in terms of anti-infection effects, improvement of microcirculatory disorders, and cardiopulmonary diuresis (24). However, the results of treatment for pediatric acute heart failure cannot be easily determined by laboratory indicators and auxiliary examinations. Instead, they can only be evaluated based on cardiac auscultation, symptoms, and changes in signs.

#### 3.2.2. Current status of combined Chinese and western medicine in treatment of acute heart failure

Timely remediation of acute heart failure can improve the cure rate and reduce morbidity and mortality. Combined treatment with Chinese and Western medicine can achieve better efficacy in remediation. Studies have shown that the use of intravenous injection of Chinese herbal medicine (Shengmai injection) and oral administration of Dushen to stabilize blood pressure can enable gradual reduction of the dose of vasoactive drugs to the point of full withdrawal, which can help to eliminate the phenomenon of dependence on vasoactive substances and to achieve better stabilization of blood pressure (25). Additionally, Chinese medicine can play an anti-inflammatory role. Some animal studies have shown that Huangqi can inhibit the activity of nuclear transcription factor IKB in myocardial tissue after infectious shock, thus playing an anti-inflammatory role, and can inhibit the production of malondialdehyde (MDA) to prevent excessive inflammatory response and immunosuppression, thus playing the role of myocardial protection. In addition to a higher resuscitation success rate, stability and long-term efficacy are also improved under this treatment (26). A clinical study showed that patients treated with a combination of Chinese and Western medicine had more successful resuscitation rates, fewer referrals for poor mental health, more stable vital signs, and better sleep and diet compared to those treated with conventional Western medicine.

### 3.3. COVID-19 sequelae and acute heart failure

**Figure 2:**
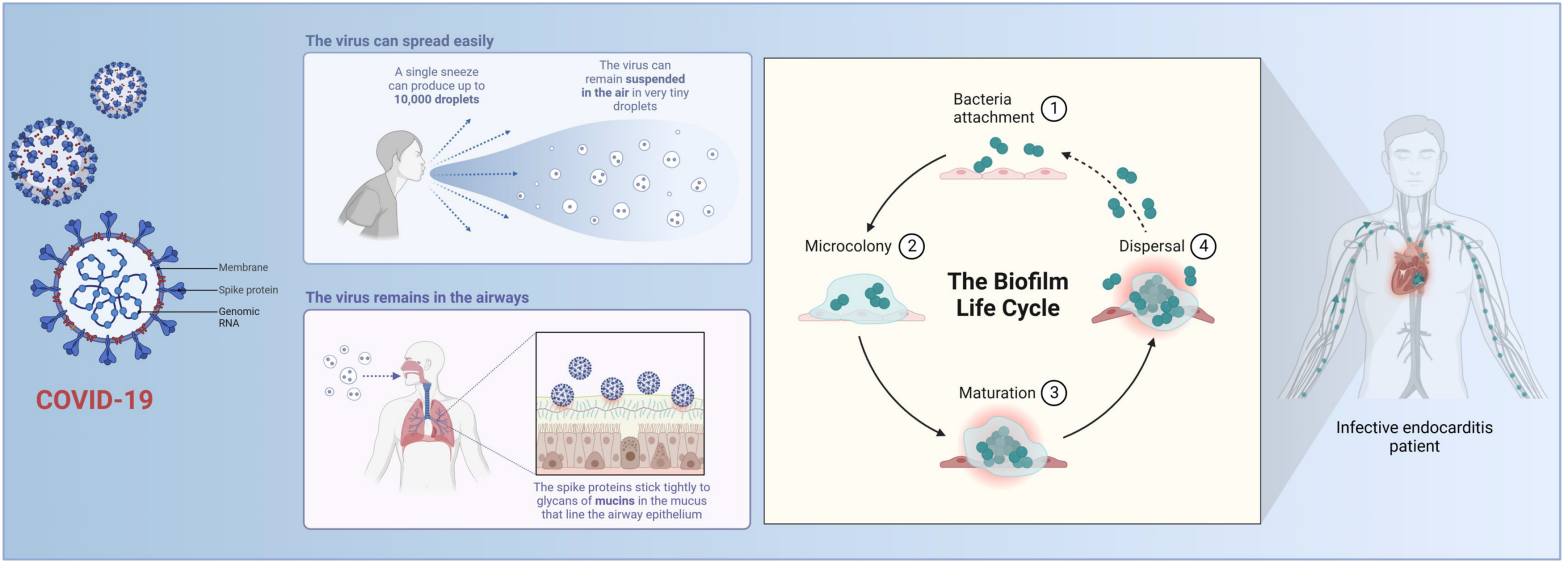
Mechanisms of COVID-19.

COVID-19 is a novel severe acute respiratory syndrome caused by a coronavirus. During the global COVID-19 pandemic, socioeconomic deprivation, social isolation, and reduced physical activity may have induced heart failure (HF) and further complications (27) (Figure 2). The pandemic has caused severe public health crises and economic hardship around the world, and has led to a surge in acute cardiovascular deaths, nearly half of which occur in the community. However, most have not been directly related to COVID-19 infection, suggesting that they are the result of delays in seeking help, psychological problems caused by COVID-19, or possibly the result of undiagnosed COVID-19 (28). It has become evident in many studies that people experience elevated levels of anxiety and depression during the acute phase, with the first appearance lasting several weeks. Technological tools, such as text messaging and online social networking platforms, can help to alleviate psychological distress in the workplace and in society (29).

COVID-19-induced HF manifests through various mechanisms, such as acute respiratory distress syndrome and respiratory failure, which can lead to acute heart failure as a result of severe hypoxia. The incidence of HF in patients with COVID-19 is significant; this is associated with a very high mortality rate, and patients with a history of HF are prone to acute decompensation after a diagnosis of COVID-19 (30). In the absence of specific drugs, the application of TCM can serve as a valuable reference treatment. Under TCM theory, the main strategies to clear the lungs, detoxify the body, and warm Yang (31). A meta-analysis in which six databases were searched between January 1 and December 31, 2020 showed that treatment with a combination of Chinese and Western medicine treatment significantly improved overall treatment efficiency, chest CT findings, disease progression, and sequelae. Thus, the combination of Chinese and Western medicine treatment is effective in improving clinical efficiency, improving clinical symptoms, and preventing disease progression in patients with COVID-19 (32).

## 4. Current status of and outlook for digital prevention and treatment of acute heart failure with Chinese and western medicine

### 4.1. Current situation

According to clinical guidelines, traditional heart failure is treated with angiotensin-converting enzyme inhibitors or angiotensin receptor antagonists, *β*-blockers, and aldosterone receptor antagonists; these treatments have apparent clinical efficacy, but they also produce adverse effects, such as individual treatment effects and drug intolerance. Chinese medicine treatment is based on the idea of diagnosis and treatment, and it is widely used in clinical treatment to provide patients with individualized treatment plans. At present, treatment of heart failure with TCM has been proven to have certain advantages in mitigating myocardial cell injury and improving cardiac function (33).

In clinical treatment, TCM combined with Western medicine is more effective than conventional Western medicine alone in the treatment of acute heart failure (34). Sildiran combined with Shenfu injection can effectively improve TCM evidence score and Lee's score; effectively improve cardiac function and hemodynamics; increase LVEF; and reduce Lac and NT-pro BNP levels (based on weekly calculations). Shenmai injection combined with recombinant human brain natriuretic peptide in the treatment of acute left-sided heart failure patients can improve the overall efficiency of treatment, improve blood gas index levels and hemodynamic indices, and reduce the incidence of adverse effects to a greater extent than recombinant human brain natriuretic peptide alone (35). The combination of Shexiangbaoxin pill and levosimendan can effectively improve heart ejection fraction and the magnitude of BNP changes, as well as reducing the incidence of adverse reactions after treatment, which can improve therapeutic effects and drug safety in the treatment of acute heart failure (36). Additionally, acupuncture at the Neiguan point combined with Western medicine has been found to achieve better efficacy in patients with acute left-sided heart failure, improving their symptoms and cardiac function; acupuncture can also effectively reduce serum myocardial injury-related markers and inflammatory cytokine levels in patients (37). Therefore, it has been confirmed that the combination of Chinese and Western medicine has improved clinical effects in treating acute heart failure and is worthy of clinical application.

### 4.2. Outlook and challenges: pain points and choke points

Prevention of acute heart failure must involve effective control of various risk factors for cardiovascular stress. Therefore, it is necessary to be able to detect and regulate physical health indicators in a timely manner in real time. An initial promising direction for the future treatment and prevention of acute heart failure is the use of digital home devices. Home device products should be visually appealing and compact in size, as well as easy and simple to operate. The risks of using them should be low and the safety value high, and other major standards in terms of product characteristics should be developed in due course in order to establish optimal quality standards for the industry, thereby avoiding variability in the quality of these products, which would result in industrial development and potential frustration. In addition, there are numerous weak links in the manufacturing of Internet of Things devices, in terms of how to achieve manufacture, the use of 3D holographic stereo laser printing equipment for processing, and the use of artificial intelligence and computing platforms that can potentially strengthen the field of modern processing technology in order to enhance effective and seamless cross-combination with traditional industrial Internet technology and enable upgrading between enterprises (38).

According to a comparison of the results of the seventh national population census and data from the 2020 China Statistical Yearbook, the census data show an increase of 14.61 million people in 2020 as compared to 2019, an increase by 0.9 percentage points. As the population ratio changes, the demand for healthcare in China will also expand in the future. There is promise and room for further development in the use of combination of Chinese and Western medicine in terms of digital treatment, intervention, and prevention of heart failure; this development will in turn drive the development of the healthcare industry and digitalization (39).

## 5. Discussion

### 5.1. Digital healthcare for acute heart failure

**Figure 3:**
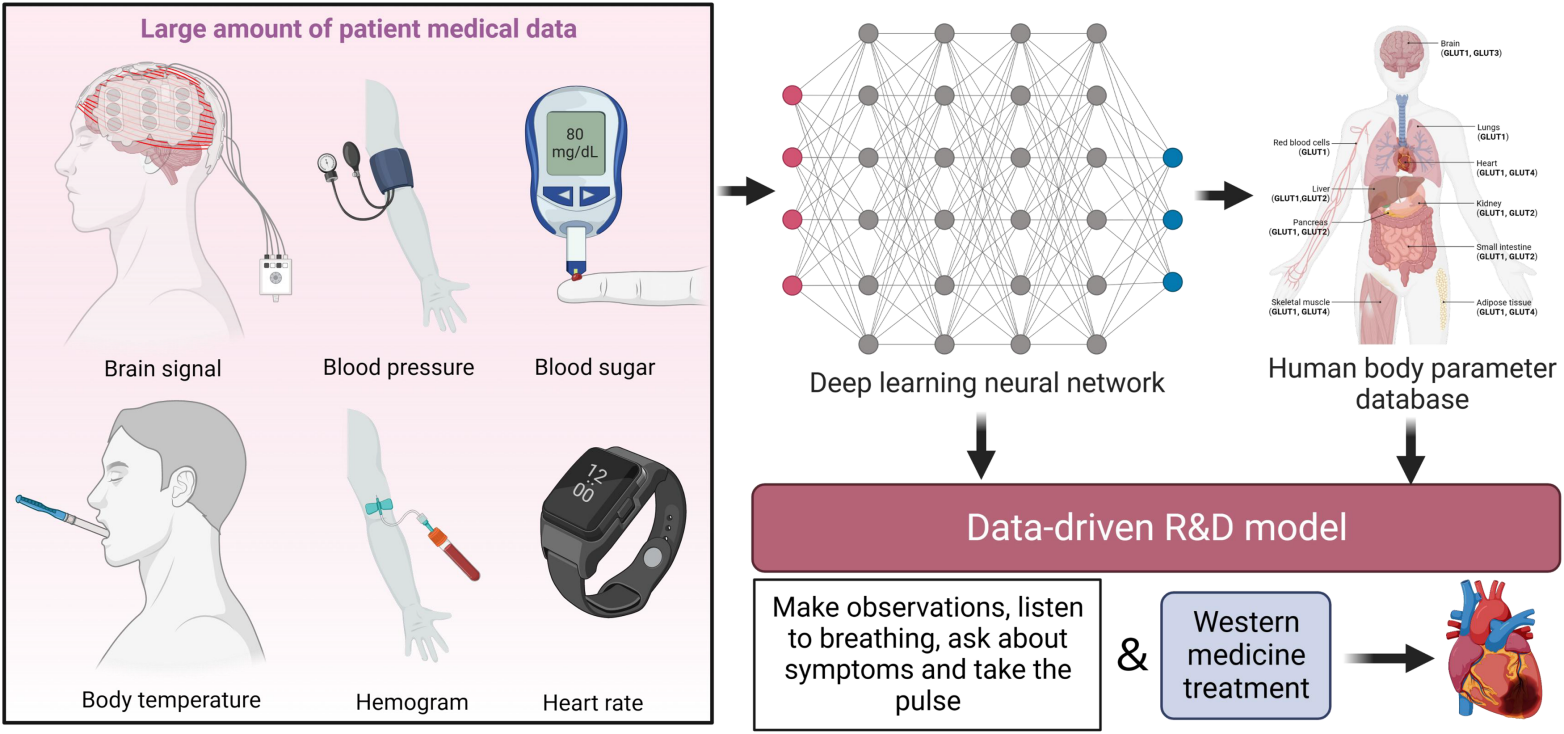
Digital prevention and treatment of acute heart failure.

Intelligent and wearable devices have moved from implants to more convenient forms, such as smart bracelets. The scope of data collection has also become more comprehensive and accuracy has improved, meaning that these devices generate a wealth of data that can support clinical decision-making and scientific research in acute heart failure (10). With the application of AI, algorithms such as machine learning and deep learning have also been introduced under digital healthcare in addition to data monitoring (12); it is worth noting that there is still tremendous room for progress in AI in the field of computer science, and that there is inconsistency and underdevelopment in the medical field in particular. In addition to algorithmic limitations, the lack of structured datasets of clinical cases of acute heart failure currently poses difficulties for data cleaning in the pre-machine learning phase, leading to mismatches in the dataset and thus the loss of large amounts of data (40). The expectation is that structured data would enable clear documentation of the condition while providing better metadata for use in digital healthcare in acute heart failure. It is also important to note that medical data is personal and sensitive data, and data security and privacy are concerns from the patient's perspective. Multiple studies have shown that many patients remain concerned about the security of their medical information and may lack confidence in the ability of current technology to protect their privacy (41). Efficient and secure storage of data will be the next challenge; the processes of using this data should also comply with the informed consent of the owner, and data should be desensitized for use in order to avoid social problems such as discrimination (42). The advancement of these processes will also involve high economic costs and problems related to the improvement of relevant laws and regulations, which need to be addressed in the context of medical science. These problems need to be solved in the domains of economics, sociology and law, and medicine (43) (Figure 3).

### 5.2. Combined treatment with Chinese and western medicine for acute heart failure

The primary treatment for heart failure is to follow the theoretical system of Chinese medicine.

Heart failure involves a combination of several pieces of clinical evidence under TCM. Heart failure is always a deficiency of the origin and a deficiency of symptoms, with qi deficiency as the primary cause and phlegm and stagnation as the symptoms, alongside yin deficiency and yang deficiency. Although there is also a typology of heart failure, the clinical reality and the symptoms described in textbooks rarely match fully, which requires TCM scholars to grasp the essence of the disease. In the process of clinical practice, the level of diagnosis is refined and experience is accumulated to identify the evidence and prescribe the appropriate treatment.

A combination of computerized and manual searches was used to search the published Chinese literature for articles published between 2005 and 2014 reporting on RCTs on the combination of enhancing Qi, warming Yang, invigorating the blood, and promoting water with Western medicine in the conventional treatment of chronic heart failure. A meta-analysis was conducted of the clinical effectiveness of herbal interventions for Yi Qi, warming Yang, invigorating the blood, and promoting water in chronic heart failure.

This analysis showed that the combination of Chinese and Western medicine has achieved further results and made further progress in the treatment of heart failure in recent years both in medical theory and on an experimental level. However, this has mostly involved the pairing of prescriptions and single herbs with the use of Western medicine; the basis for the validation of the mechanisms underlying the use of drug pairings and empirical prescriptions needs to be further explored. At the same time, in clinical work, efforts should be made in the direction of high efficiency, the use of trace amounts of drugs, and medications that are easy to take, while ensuring efficacy and taking into account the different dosages of particular drugs that patients in different conditions can take.

### 5.3. Digital prevention and treatment of acute heart failure through a combination of Chinese and western medicine

There are many ideas for how to combine Chinese and Western medicine in the treatment of acute heart failure in Chinese and Western hospitals, and a great deal of consensus; rich clinical experience has also been acquired and great achievements made in digital prevention and treatment of acute heart failure. There is an urgent need for a new system of prevention and treatment that can combine the objective diagnosis and treatment techniques of Western medicine with the unique and practical experience in prevention and diagnosis of Chinese medicine, and with digital tools such as artificial intelligence.

The combined empirical knowledge and diagnostic scope of Chinese and Western medicine in acute heart failure is characterized by certainty, completeness, and objectivity, ensuring the capacity of machine learning algorithms to understand the relevant reasoning and providing robust data support for it; this is conducive to the establishment of a knowledge map. Therefore, the use of cognitive systems combining knowledge of Chinese and Western medicine for specialized treatment of certain diseases is a possible path for artificial medical intelligence and a future trend in development (44). Digital healthcare also has the advantages of case efficiency, affordability, and scalability. Recent work suggests that audio-based methods can be used for research (45). However, lack of awareness of bias and methodological decision-making may affect these tools' performance in practice. The greatest role played by artificial intelligence appears to be in diagnostic imaging, such as analysis of CT scans or chest x-ray images. C-reactive protein, white blood cell count, creatinine, lactate dehydrogenase, lymphocyte discovery, and platelet count (46). However, there is far more scope for technological advancement than we might think. Under the influence of the COVID-19 pandemic, along with significant trends in data analysis in various countries, the deployment of prevention and treatment methods involving the integration of traditional Chinese and Western medicine, as described above, represents a potential approach to further improve the efficiency of public health measures.

In addition to the use of the wearable devices mentioned above, at the diagnosis and treatment level, the diagnosis method in traditional Chinese medicine is to combine four different diagnostic processes. The information obtained from these is used as the basis for diagnosis and treatment; specifically, the four diagnostic processes used by practitioners of Chinese medicine are making observations, listening to the patient's breathing, asking about their symptoms, and taking their pulse using the fingers (47). However, since this process relies primarily on the experience of physicians, and the level of medical and clinical experience varies among individual physicians and lacks a quantified standard, a four-diagnosis instrument has been developed to replace this process. The form of this four-diagnosis instrument, as currently developed, is a simple imitation of the four diagnostic processes of TCM; it is not based on clinically valid case data, and it has certain limitations. The diagnostic results for the four diagnostic processes are based entirely on a database of TCM diagnoses, without the use of an intelligent process involving continuous iteration of machine learning; additionally, the rate of correct diagnoses is fixed and cannot be improved due to problems with the algorithm, which is entirely different from the “black-box” diagnostic process of TCM as simulated by artificial intelligence. The superior performance of AI technologies, such as deep learning, relies on the use of a data-driven R&D model, which does not require pre-built *a priori* models. This approach falls under the category of a “black/gray-box” model, which applies AI-aided diagnosis using a combination of Chinese and Western medicine, whereas the diagnostic decision-making system in Chinese medicine is considered to be a “black box”; this is expected to become a “gray box” with the help of AI. This article has described the possibility of the use of a combination of Chinese and Western medicine, alongside digitalization, to create a new digital model for prevention and treatment.

## 6. Conclusion

With rapid advances in experimental technology and computer science, digital medicine and the integration of Western and Chinese medicine will represent the future of healthcare. Access to better healthcare, digital healthcare technologies such as telemedicine, and remote monitoring have the potential to bring healthcare to underserved and remote populations. Patients can connect with medical professionals from the comfort of their homes, saving time and money and reducing the burden of transportation. Patients can track their symptoms, monitor their medications, and communicate with their healthcare providers in real time in order to better self-manage and adhere to their treatment plans. At the same time, digital health technologies can assist healthcare professionals in their daily clinical practice, improving the accuracy and speed of diagnosis and treatment. Machine learning algorithms, for example, can analyze large amounts of patient data, thus enabling personalized treatment with a combination of Western and Chinese medicine. All of this requires the effective use of medical information, frameworks for digital technology, artificial intelligence models, and medical devices in order to ensure improved healthcare outcomes. This approach also provides a basis for the development of new strategies and protocols for the clinical management of acute heart failure.
